# Computational Assessment of Protein–protein Binding Affinity by Reversely Engineering the Energetics in Protein Complexes

**DOI:** 10.1016/j.gpb.2021.03.004

**Published:** 2021-04-07

**Authors:** Bo Wang, Zhaoqian Su, Yinghao Wu

**Affiliations:** Department of Systems and Computational Biology, Albert Einstein College of Medicine, Bronx, NY 10461, USA

**Keywords:** Protein–protein interaction, Binding affinity, Non-interfacial residue, Knowledge-based potential, Monte-Carlo simulation

## Abstract

The cellular functions of proteins are maintained by forming diverse complexes. The stability of these complexes is quantified by the measurement of **binding affinity**, and mutations that alter the binding affinity can cause various diseases such as cancer and diabetes. As a result, accurate estimation of the binding stability and the effects of mutations on changes of binding affinity is a crucial step to understanding the biological functions of proteins and their dysfunctional consequences. It has been hypothesized that the stability of a protein complex is dependent not only on the residues at its binding interface by pairwise interactions but also on all other remaining residues that do not appear at the binding interface. Here, we computationally reconstruct the binding affinity by decomposing it into the contributions of interfacial residues and other **non-interfacial residues** in a protein complex. We further assume that the contributions of both interfacial and non-interfacial residues to the binding affinity depend on their local structural environments such as solvent-accessible surfaces and secondary structural types. The weights of all corresponding parameters are optimized by **Monte-Carlo simulations**. After cross-validation against a large-scale dataset, we show that the model not only shows a strong correlation between the absolute values of the experimental and calculated binding affinities, but can also be an effective approach to predict the relative changes of binding affinity from mutations. Moreover, we have found that the optimized weights of many parameters can capture the first-principle chemical and physical features of molecular recognition, therefore reversely engineering the energetics of protein complexes. These results suggest that our method can serve as a useful addition to current computational approaches for predicting binding affinity and understanding the molecular mechanism of **protein–protein interactions**.

## Introduction

Most of the biological processes in cells are maintained by interactions between different proteins [1], [2], [3], [4], [5], [6]. Whether two specific proteins interact and how stable the interaction is are largely determined by the three-dimensional (3D) structures of these molecules, especially at the interface of the complex [7]. The stability of a complex that is formed between two proteins can be quantified by their binding affinity. Traditionally, the binding affinity is described by the experimental measurement of the equilibrium dissociation constant *K_d_*. It is related to the change of Gibbs free energy after binding (ΔG) through the equation $\Delta G=RT\;\ln\;K_{d}$ [8]. Numerous studies have shown that various diseases such as cancer and diabetes originate from mutations that alter the binding affinity of interacting proteins [9], [10], [11]. Therefore, accurate estimation of the binding affinity and the effects of mutations on changes of binding affinity is crucial to understanding the biological functions of proteins and their dysfunctional consequences.

Binding affinity can be determined by various experimental techniques including isothermal titration calorimetry (ITC) [12] and surface plasmon resonance (SPR) [13]. However, measuring binding affinity with these methods is a time-consuming and labor-intensive process, not mentioning the intrinsic limitations associated with these approaches [14]. For instance, ITC cannot be used for measuring protein–protein interactions (PPIs) with very low or high affinities [15], and the accuracy of measured association rates in SPR is debatable due to the fact that the immobilization of interacting proteins on the sensor surface can affect the conformational and rotational entropy of binding [16]. Relative to these traditional experimental approaches, predicting binding affinity by computational methods is not only less time-consuming and labor-intensive but can also unravel the molecular mechanism of PPIs with details that are inaccessible through experimental measurements. As a result, more computational methods have been developed to predict the binding affinity or mutation-induced affinity changes of PPIs [17], [18], [19], [20], [21], [22], [23], [24], [25], [26], [27], [28]. For instance, scoring functions based on distance-scaled, finite ideal-gas reference state (DFIRE) [29], RosettaDock [30], and ATTRACT [31] force fields have been applied to calculate the binding affinities for protein complexes. However, by training and testing different machine learning algorithms, a large set of molecular descriptors has been constructed to calculate the binding affinity [22]. The correlation coefficients between the binding affinities predicted by these computational approaches and their corresponding experimental data range from 0.4 to 0.75. Although most of these methods focus on the physical and chemical properties at the binding interfaces of protein complexes, recent studies have also demonstrated the importance of non-interfacial residues in regulating the affinity of protein binding [6].

Here, we computationally assessed the stability of a protein complex by decomposing the binding affinity into the contributions of interfacial residues and other non-interfacial residues in the complex. The energetic contributions of both interfacial and non-interfacial residues in our model are related to the structural features of interacting proteins, such as secondary structural types and solvent-accessible surfaces (SASs). We used the Monte-Carlo algorithm to search the parameter space of these energetic contributions and optimize the weights of the parameters. Our model was tested against Structural database of Kinetics and Energetics of Mutant Protein Interactions (SKEMPI), a large-scale database that contains 158 wild-type protein complexes and 3048 associated mutants with experimentally determined *K_d_* and available 3D structures [32]. After cross-validation, we showed that there was a strong correlation between the experimentally measured binding affinities and our calculated values. While taking a closer look at the values of weight in the optimized parameter space, we found that many captured the first-principle chemical or physical characteristics of biomolecular recognition, therefore reversely engineering the energetics of protein complexes. These results suggest that our method is not only a useful computational tool for predicting binding affinity but also provides new insights for understanding the molecular mechanism of PPIs.

## Method

### Knowledge-based potential from a database of protein complexes

A knowledge-based potential is first constructed to evaluate the energies of protein complexes. The potential gives the energetic parameters for all combinations of residue pairs that appear at the protein binding interface. Specifically, the binding energy $u\left(i,j\right)$ between residue types *i* and *j* can be denoted as follows:

(1) $$u\left(i,j\right)=-kT\ln\frac{N_{\mathrm{obs}}\left(i,j\right)}{\chi_{i}\chi_{j}N_{\mathrm{obs}}}$$

where $N_{\mathrm{obs}}\left(i,j\right)$ is the number of observed pairs between residue types *i* and *j* at the protein binding interface [33], $N_{\mathrm{obs}}$ is the number of total residue pairs at the protein binding interface, and *χ_i_* is the mole fraction for residue type *i* at the interface. Two residues form a contact if the distance between any atoms from the sidechains of these residues is less than the defined cut-off distance (5.5 Å).

The specific values of Eq. (1) are calculated by counting the corresponding residue pairs observed in a large-scale, structure-based protein complex library. The three-dimensional interacting domains (3did) database is used to construct the library [34]. The inter-domain interactions are collected in the 3did database for all protein complexes, if their 3D structures are available in high resolutions. The basic unit of the database is an item called “interacting domain pair” (IDP). Each IDP could be either a homodimer or a heterodimer within a single protein complex. It could also be an inter-domain interaction within a multi-domain protein. The Pfam index is given to both domains of an IDP [35]. Each IDP further consists of various numbers of instances. The specific instances are known as 3D items, in which information [*e.g.*, Protein Data Bank (PDB) index, residue range, and chain ID] is provided to both interacting protein domains. From each IDP in the 3did database, only one representative 3D item is selected to reduce sequence redundancy in the library. As a result, there are 4960 entries of homodimeric or heterodimeric PPIs in the final library.

### Reformulating the binding affinity based on an *ab initio* procedure

We hypothesize that the stability of a protein complex is dependent on both the residues at the binding interface by pairwise interactions and all other remaining residues that do not appear at the binding interface. These non-interfacial residues could be either on the surfaces or in the interiors of the protein complex. Moreover, the contributions of both interfacial and non-interfacial residues to the binding affinity are dependent on their local structural environments such as the secondary structural types and SASs. Consequently, the binding affinity between protein *A* and protein *B* can be described as follows:

(2) $$\begin{array}{cc} \Delta G\left(A,B\right) & =\underset{i,j}{\sum }W_{\mathrm{itf}}\left({aa}_{i}^{A},{ss}_{i}^{A},aa_{j}^{B},{ss}_{j}^{B}\right)+w_{n}\times \\ & [\sum_{i}W_{\mathrm{nitf}}\left({aa}_{i}^{A},{ss}_{i}^{A},SAS_{i}^{A}\right)\\ & +\sum_{j}w_{\mathrm{nitf}}\left({aa}_{j}^{B},{ss}_{j}^{B},{SAS}_{j}^{B}\right)] \end{array}$$

In Eq. (2), the first term on the right side is summarized over all pairs of residues that form contacts at the binding interface of the protein complex. If the distance of any atom from the sidechain of residue *i* and any atom from the sidechain of residue *j* is smaller than the cut-off value of 5 Å, these two residues form a contact at the binding interface [36], [37], [38]. Here, residue *i* and residue *j* belong to different proteins in a complex. The weight of this interfacial energy term not only depends on 20 different types of amino acids $aa_{i}^{A}$ and $aa_{j}^{B}$, but is also sensitive to the secondary structural elements $SS_{i}^{A}$ and $SS_{j}^{B}$ of interfacial residues *i* and *j*. The secondary structure of a residue is determined by the standard DSSP algorithm [39] with three categories: helix (H), strand (S), and loop (L). In contrast, the second and third terms on the right side of Eq. (2) are summarized over all non-interfacial residues of proteins *A* and *B*, respectively. The weights of these non-interfacial energy terms not only depend on 20 different types of amino acids and 3 different types of secondary structural elements, but also are sensitive to the SAS for each residue in the corresponding protein. The Shrake-Rupley algorithm is applied to calculate the SAS of a residue [38], in which the radius of a water molecule probe is 1.4 Å and the values of van der Waals (VDW) radii for all atom types are taken from previous literature [38]. Based on the calculated SASs, non-interfacial residues are classified into two groups: buried (B) and exposed (E). A residue is exposed if more than 30% of its sidechain surface is solvent-accessible, otherwise, the residue is considered buried. Finally, the value of *w_n_* is used to scale the relative contributions between interfacial and non-interfacial residues.

### Weight refinement by a Monte-Carlo-based algorithm

To retrieve the energetic contributions of PPIs and accurately calculate their binding affinities, the sampling of parameter space in Eq. (2) is carried out by a Monte-Carlo procedure. The weights of all parameters in Eq. (2) are optimized by maximizing the correlation coefficient between the calculated and experimental binding affinities. Given the data of binding affinities calculated by Eq. (2) and their experimental counterparts, the correlation coefficient *ρ* is calculated as follows:

(3) $$\rho \left(\mathrm{calc},\exp\right)=\frac{\overset{N}{\underset{i=1}{\sum }}\left(\Delta G_{\mathrm{calc}}^{i}-\Delta {\overset{¯}{G}}_{\mathrm{calc}}\right)\times \left(\Delta G_{\exp}^{i}-\Delta {\overset{¯}{G}}_{\exp}\right)}{\sqrt{\overset{N}{\underset{i=1}{\sum }}{\left(\Delta G_{\mathrm{calc}}^{i}-\Delta {\overset{¯}{G}}_{\mathrm{calc}}\right)}^{2}}\times \sqrt{\overset{N}{\underset{i=1}{\sum }}{\left(\Delta G_{\exp}^{i}-\Delta {\overset{¯}{G}}_{\exp}\right)}^{2}}}$$

where *N* is the total number of protein complexes used in the refinement. The variable $\Delta G_{\mathrm{calc}}^{i}$ is the binding affinity of the *i*-th protein complex calculated by Eq. (2), while $\Delta G_{\exp}^{i}$ is the experimental binding affinity for the corresponding protein complex. Relatively, $\Delta {\overset{¯}{G}}_{\mathrm{calc}}$ is the average value of calculated binding affinities over all protein complexes, while $\Delta {\overset{¯}{G}}_{\exp}$ is the average value of experimental binding affinities over all protein complexes.

The refinement begins from an initial condition in which all parameters are given random weights ranging between −1 and 1. A Monte-Carlo movement is applied by randomly selecting a parameter with equal probability in the parameter space and updating the weight of the selected parameter by a different new value among the same range between −1 and 1. The correlation coefficients are calculated before and after the movement. The updated weight is accepted if the correlation is higher. Otherwise, the movement is rejected, and the weight of the selected parameter maintains its original value. The system is iterated by a series of such Monte-Carlo steps until the calculated correlation coefficient becomes stabilized or a pre-defined number of simulation steps is reached. Finally, to avoid overfitting during the refinement, cross-validation is applied to test the robustness of the procedure. This procedure is described in the next section.

### Dataset used in the benchmark test

The experimental values of binding affinity used in this study are derived from the SKEMPI database [32]. The SKEMPI database is a comprehensive database that contains experimental data not only on the absolute values of binding constants for wild-type protein complexes but also on the changes of these values upon mutations. It includes data for 158 wild-type protein complexes and their 3048 associated mutants. Most binding constants are experimentally determined using SPR, ITC, and various other spectroscopic methods such as stopped-flow fluorescence. They are collected from many studies including systematic mutation scans, site-directed experiments, cognate/non-cognate pairs, homolog scanning mutagenesis, and interface engineering studies. Moreover, the structures of all wild-type complexes in the database have been solved and are available in the PDB. These data are available online (https://life.bsc.es/pid/mutation_database/).

Other databases also contain information on protein complex structures and measured binding affinities. For instance, Kastritis et al. [14] built a benchmark set for protein–protein binding affinity that contains 144 complexes with available high-resolution structures. Another docking and affinity benchmark set developed by Vreven et al. [40] includes 179 entries of experimentally measured binding affinities. These datasets and the SKEMPI database heavily overlap. More importantly, only the SKEMPI database systematically lists information about the relative changes of binding affinities due to mutagenesis. Finally, it is worth mentioning that very recently, the SKEMPI database has been upgraded to its 2.0 version [41]. The new dataset will be integrated into our method to update the parameters in the future.

### The detailed strategy of leave-one-out cross-validation

The Monte-Carlo-based refinement is combined with a leave-one-out cross-validation strategy to test whether the optimized weight parameters can predict binding affinities for unknown protein complexes. The cross-validation contains 158 separate runs, and each run further consists of independent refinement and testing phases. During each run of cross-validation, one of the 158 wild-type protein complexes, together with its associated mutants, is selected for testing, while the remaining data in the SKEMPI database are used for refinement. There are always more than one data point in the testing set. In addition to the binding affinity of the wild-type protein complex, each testing set also contains the binding affinities of various mutants, in which one or multiple residues of the complex are replaced by other types of amino acids. For instance, the testing set for the Barnase/Barstar complex (PDB ID: 1BRS) includes 95 different mutants, so there are 96 data points in total.

Given the organization of the datasets, cross-validation is conducted as follows. In the refinement phase, the values of the parameters in Eq. (2) are optimized by the Monte-Carlo simulations to maximize the correlation between calculated and experimental binding affinities. The optimized weight parameters are then used to calculate the binding affinities of protein complexes that are left in the testing set. The final output of calculated binding affinities for the tested protein complexes is adjusted according to the average value and standard deviation (SD) of the binding affinities included in the refinement. After the entire procedure of cross-validation, the values of binding affinities for all 158 wild-type protein complexes and their associated mutants in the SKEMPI database can be calculated and compared with their corresponding experimental measurements.

### Definition of sensitivity, specificity, precision, and accuracy of prediction results

Sensitivity is defined as the percentage of correct predictions among all mutants in the dataset that increase binding affinity. Specificity is defined as the percentage of correct predictions among all mutants in the dataset that decrease binding affinity. Precision is defined by calculating the ratio between the number of mutants that are correctly predicted to increase binding affinity *versus* the total number of predictions that are reported to increase binding affinity. Finally, the overall accuracy is defined by calculating the ratio between the number of mutants that are correctly predicted to either increase or decrease binding affinity *versus* the total number of mutants in the dataset.

## Results and discussion

### Calculating the binding energy of PPI by knowledge-based potential

**Figure 1:**
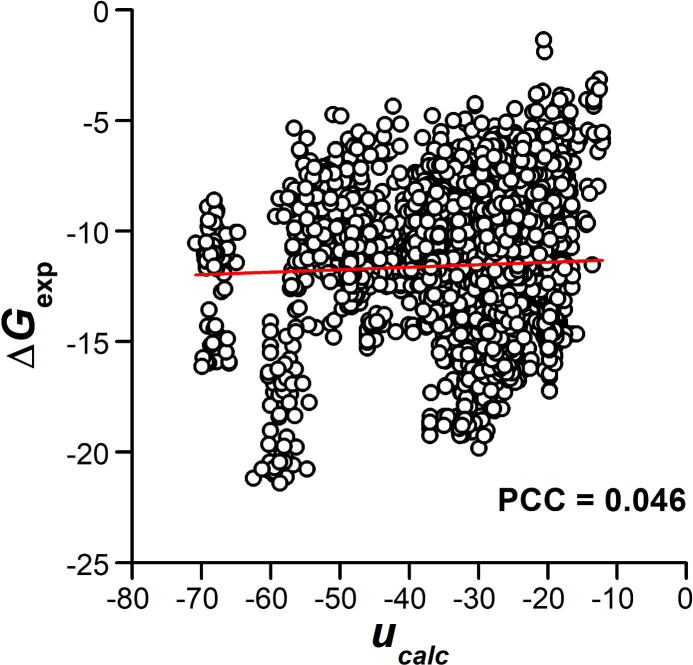
**Comparison of the binding energy of PPI calculated by knowledge-based potential with the experimentally measured binding affinity** The binding energies calculated by knowledge-based potential were compared with the experimentally measured binding affinities for all protein complexes in the SKEMPI database. PPI, protein–protein interaction; *u_calc_*, binding energy calculated by knowledge-based potential; Δ*G*_exp_, experimentally measured binding affinity; PCC, Pearson correlation coefficient.

We first assumed that the stability of a protein complex could be determined by adding the interactions between residues across the binding interface. Based on the residue-based interactions derived from the statistics of structurally available protein complexes, we calculated the binding energy of a protein complex using our constructed knowledge-based potential. The potential gives the energetic parameters for all types of residue pairs at the binding interface, and the calculated binding energy of a protein complex with a known 3D structure is the summation of all residue pairs that form contacts at the interface (see Method for details). We compared our calculated binding energies with the experimentally measured binding affinities for all protein complexes in the SKEMPI database. As shown in Figure 1, no obvious correlation [Pearson correlation coefficient (PCC) = 0.046] was observed between the statistical-based binding energies and experimental binding affinities.

The reason for this low correlation is mainly due to the fact that not all residues at the binding interface play an equal role in regulating affinity. Certain critical residues at the binding interface, called hot spots, contribute most to binding [42]. However, while deriving the knowledge-based potential using Eq. (1), a common assumption is that all pairs of residues at the binding interface of a protein complex contribute equally to the total energy of the complex. In other words, the knowledge-based potential can only provide the likelihood of all residues appearing at the binding interface of a native protein complex without offering their relative contributions to affinity. Moreover, mutations of residues that are remote from the binding interface can cause differences in the total binding affinity. The energetic contributions of these non-interfacial residues are not accounted for in the knowledge-based potential. Therefore, the factors for evaluating binding affinity need to be revisited.

### Reconstructing the binding affinities of protein complexes by weight optimization

Given that the stability of a protein complex cannot be directly reflected by the parameters derived from statistical potential, we reconstructed the calculation of binding affinity by Eq. (2) (see Method for details). We optimized the weights of all energetic terms in the equation by a Monte-Carlo-based algorithm and tested the optimized weights through the leave-one-out strategy of cross-validation. In brief, the dataset was divided into two groups for refinement and testing. The weights were tuned by maximizing the correlation between the calculated and experimentally measured binding affinities in the refinement. These weights were then used to predict the binding affinities of protein complexes in the testing sets. We reorganized the testing sets by order so that by the end of the cross-validation, the binding affinities for all protein complexes in the dataset were tested. The refinement and cross-validation processes are described in more detail in Method.

**Figure 2:**
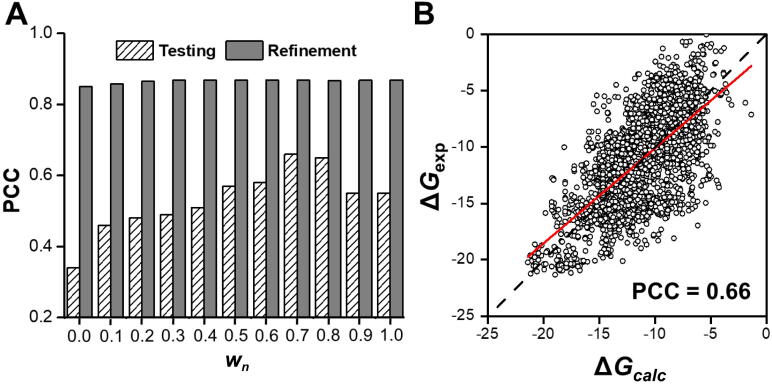
**Reconstruction of the binding affinity based on weight optimization** **A.** Bar chart showing the refinement and testing PCCs under different values of *w_n_*. *w_n_* indicates the weight between the interfacial and non-interfacial energetics. **B.** Comparison of calculated binding affinities with experimental measurements for all 3205 protein complexes in the dataset under the optimal *w_n_* of 0.7. Each dot represents a specific protein complex. The coordinates along the x-axis give the predicted values of binding affinity using the weights that are optimized during refinement, and the coordinates along the y-axis give the experimental values.

The value of *w_n_* was first changed to investigate the relative impacts of interfacial and non-interfacial residues on binding affinity. In detail, we increased *w_n_* from 0 to 1 and carried out the same refinement and testing procedures during this process. For each specific value of *w_n_*, we calculated the maximized PCC after refinement as well as the predicted PCC for testing. Figure 2A shows both the refinement and testing PCCs under different values of *w_n_*. Not surprisingly, the PCCs calculated for refinement were always higher than those for the actual prediction. Moreover, the testing results improved when *w_n_* was increased from 0. The best performance, however, was only obtained when *w_n_* fell into a small range between 0.7 and 0.8. The testing PCCs of prediction reached the highest values within this range. Further increases of the contribution from non-interfacial residues, however, caused negative effects on the testing PCCs. These results indicate that the energetics of non-interfacial residues positively contribute to the binding affinity, and that this contribution is balanced by the inter-molecular interactions at the binding interface of a protein complex.

Given the optimal weight between the interfacial and non-interfacial energetics (*w_n_ =* 0.7), we compared our calculated binding affinities with the experimental measurements for all 3205 protein complexes in the dataset. As shown in Figure 2B, there was a strong correlation between the experiments and our calculations (PCC = 0.66) while accounting for all protein complexes. This result indicates that the binding affinity of a protein complex can be captured by the parameters described in Eq. (2).

### Testing the statistical significance of the prediction results

**Supplementary Figure S1:**
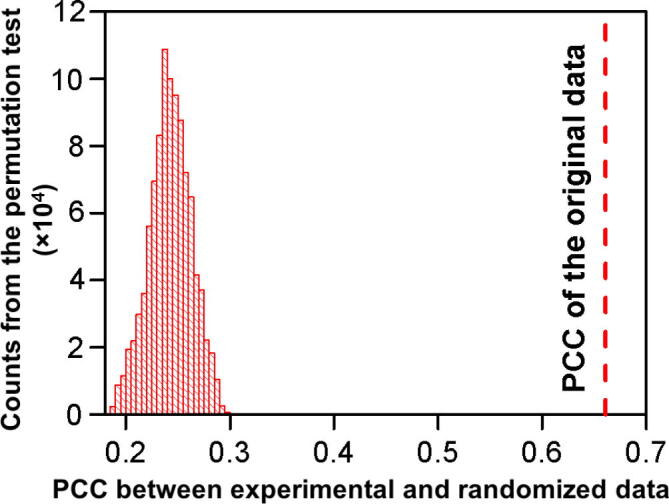
**The comparison of our prediction results with a simple null model** The Pearson correlation coefficient values (red histogram) were calculated from the permutation tests and compared with the original data (red dashed line). The p-value was calculated from the distribution to reject the null hypothesis, in which we assume there is no correlation between experimental and our calculated binding affinities.

To assess the statistical significance of the derived correlation between the calculations and experimental data, we first tested our results against a null hypothesis in which the experimental and calculated data show no correlation. We applied the permutation tests for the null hypothesis. In the permutation tests, the experimental binding affinities were fixed, and the calculated binding affinities were randomly reshuffled. The PCC was then calculated between the experimental and randomized data. This process was repeated 1 × 10^6^ times. Finally, the *P* value was calculated as the proportion of PCCs generated in the permutation tests that are larger than the PCC of the original data. As a result, none of the 1 × 10^6^ PCCs from the randomized data were larger than the original PCC (0.66). The distribution of these PCCs is plotted in Figure S1. Therefore, the null hypothesis was rejected with a *P* value lower than 1 × 10^−6^, and the alternative hypothesis was accepted, *i.e.*, the correlation between the experimental and calculated data was significant.

To further estimate the confidence interval of our calculated PCCs, we conducted 10 runs of cross-validation. The leave-one-out test was performed during each run. The weight between the interfacial and non-interfacial energetics *w_n_* was fixed at 0.7. All other weights were first refined by Monte-Carlo optimization, and the binding affinities were then calculated against the testing sets, as described above. The final testing PCC was obtained after completing the cross-validation for the entire dataset. This procedure was repeated 10 times. The average value and SD of the PCCs were calculated. The PCCs obtained during refinement and testing for each run of cross-validation are listed in Table S1. The average value of the PCCs was 0.66, and the SD was 0.017. Therefore, the overall accuracy of the derived PCC between the experimental and calculated binding affinities was 0.66 ± 0.017, with a confidence level of 0.95.

### Estimating the absolute values and mutation-induced relative changes of binding affinity

**Figure 3:**
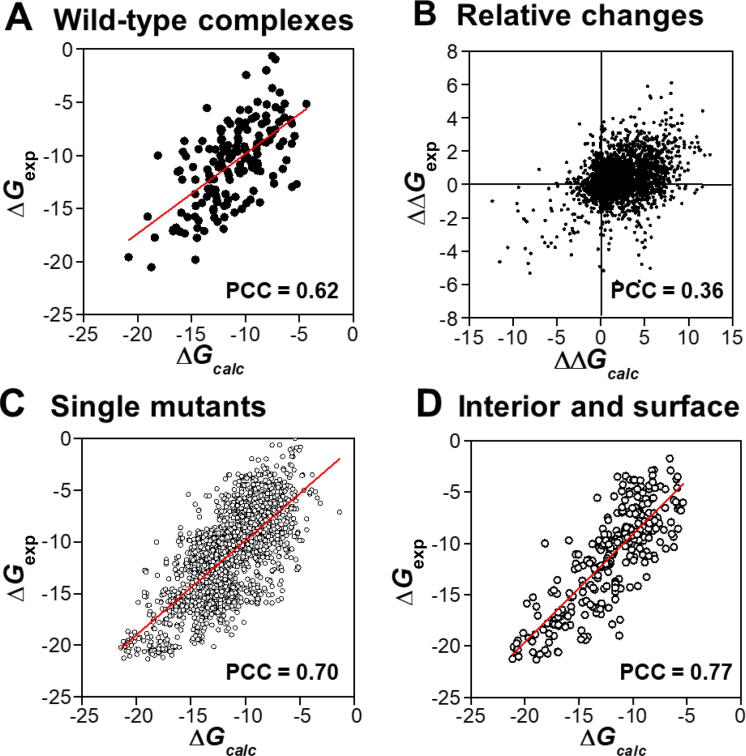
**Detailed analyses of the correlations between our calculations and experimental data under various conditions** **A.** Comparison of the absolute binding affinities of all 158 wild-type complexes in the SKEMPI database with their relative experimental data. **B.** Comparison of the relative changes of binding affinities for all mutants with their relative experimental data. **C.** Comparison of the calculated binding affinities of all 2178 single mutants with their relative experimental data. **D.** Comparison of the calculated binding affinities of the single mutants belonging to the “surface” and “interior” groups with their relative experimental data.

The dataset contains both wild-type protein complexes and complexes with one or more mutant residues. To assess whether our computational model is able to estimate the absolute binding affinities of wild-type complexes and/or their relative changes due to mutations, we separated 158 wild-type protein complexes from the dataset and identified 3048 mutants corresponding to these wild-type complexes. We calculated the absolute binding affinities for all wild-type complexes as well as the relative changes of binding affinities for all mutants, and then compared them with the experimental data. As shown in Figure 3A, our model is accurate in calculating the absolute binding affinities for all 158 wild-type protein complexes (PCC = 0.62). Figure 3B shows the relative changes of binding affinities that were detected experimentally *versus* our prediction for all mutants in the dataset. The relative change of binding affinity for a mutant was derived by calculating the difference between the absolute binding affinity of this mutated complex and that of its wild-type counterpart.

Figure 3B not only shows a positive correlation (PCC = 0.36) but also contains more quantitative information. Based on the calculation of free energy changes, we can make a binary prediction of whether a specific mutation effectively increases or decreases the binding affinity relative to the wild-type protein complex. To further calibrate our prediction, we calculated the sensitivity, specificity, precision, and accuracy from the testing results after cross-validation. Based on the definition described in Method, we found that our prediction could achieve a total sensitivity of 0.61, specificity of 0.56, precision of 0.84, and overall accuracy of 0.56. Taken together, these results demonstrate that our computational model can effectively predict the binding affinities of protein complexes on both absolute values and their relative changes induced by mutations.

### Evaluating the effects of mutations at different locations of protein complexes

**Figure 4:**
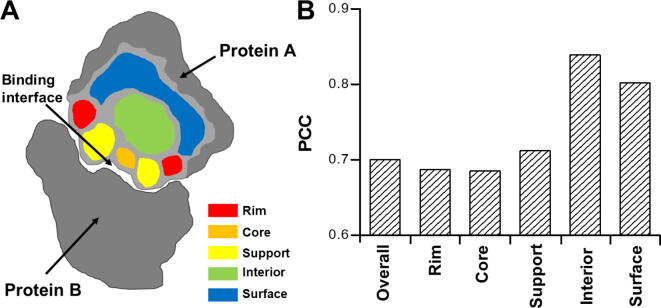
**Effects of mutations at different locations of protein complexes** By comparing the SAS of a residue in the monomer to that in the complex, we divided a protein complex into five different structural regions, which are coded by different colors in (**A**). We classified all single mutants in the SKEMPI database into five groups according to these definitions. We calculated the PCCs by comparing the calculated binding affinities with experimental values for these five groups (**B**). SAS, solvent-accessible surface.

Mutations might occur at any location of a protein complex. They affect the binding affinity even if they are not directly involved in the binding interface. To study the sensitivity of our model in calculating the changes of binding affinity to different locations of mutants, we followed the definition of a previous study [43], *i.e.*, dividing a protein complex into five structural regions (Figure 4A) by comparing the SAS of the residue in the monomer to that in the complex. The interfacial residues were differentiated into three sections: core, rim, and support, while the remaining parts were classified as either “surface” or “interior”. To avoid further complication, only single mutants in the SKEMPI database were considered in the analysis, because multiple mutants could be found simultaneously in different regions of a protein complex. Overall, the PCC between our calculated binding affinities and the experimental values for 2178 single mutants was 0.70 (Figure 3C), which was slightly better than the correlation for all data.

We further decomposed all single mutants into five groups by the aforementioned definition of structural regions and calculated the PCCs between the calculated and experimental binding affinities for these groups (Figure 4B). The results showed that the effects of mutations on binding affinity were closely related to the spatial locations of residues in the complex. In addition, our predictions were more accurate in the “surface” (PCC = 0.80) and “interior” (PCC = 0.84) groups, in which the mutations of residues are not located at the binding interfaces of protein complexes. Moreover, a high correlation (PCC = 0.77) was observed between the calculated and experimental data for the single mutants belonging to both groups (Figure 3D). The high correlation between the calculated and experimental binding affinities for mutants at non-interfacial regions was due to the specific incorporation of the energetic contributions of non-interfacial residues in our computational model. Moreover, these contributions depend on the local structural environments of each residue in protein complexes. Therefore, the difference of mutations for residues at either the surface or interior of a protein can be distinguished.

### Reversely engineering the energetics in protein complexes

The SKEMPI database contains 158 native protein complexes. As described in Method, during the leave-one-out cross-validation, one of these native protein complexes, together with all associated mutants, was excluded from refinement for testing purpose, while the remaining data in the SKEMPI database were used to optimize the weights. As a result, the cross-validation included 158 runs of the individual refinement process. All refinement processes were independent from each other, and each run led to a set of different values at the end. To test if these optimized parameters are biologically meaningful, we calculated the average values for all weights in Eq. (2) and their associated SD values over all runs of refinement. Detailed values of all parameters are listed in Tables S2 and S3. The relatively small distributions of SD values for most parameters suggest that the refinements of different runs are all converged to a similar result. This indicates that the overall procedure of cross-validation is stable, and the derived values of weights are not chosen randomly.

**Figure 5:**
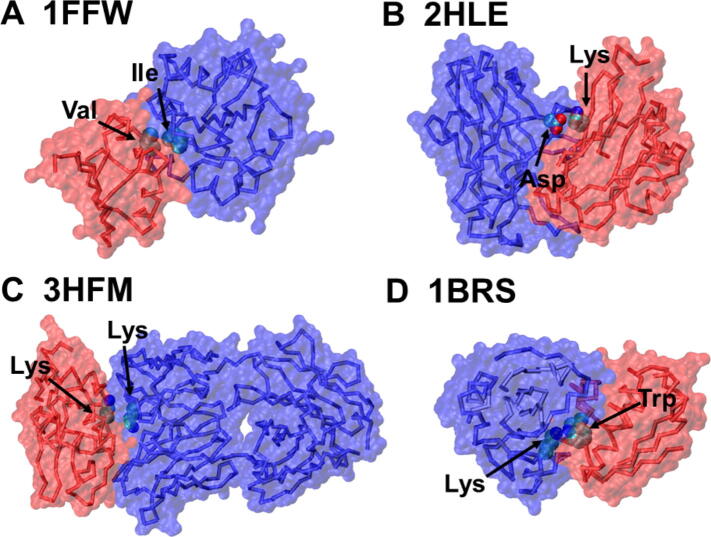
**Some examples for pairwise amino acids with the lowest values of interfacial parameters** Specifically, the hydrophobic effect between Ile and Val plays an important role in regulating the stability of protein complexes (**A**). Amino acids with opposite charges (*e.g.*, between Lys and Asp) provide important electrostatic contributions for binding affinity (**B**). We also found that positively charged residues (such as Lys) have an average tendency to pair among each other (**C**). Finally, when a cationic sidechain (such as Lys) is near an aromatic sidechain (such as Trp), they are stabilized by a cation-π interaction (**D**). The PDB ID of each protein complex is indicated on the top of each panel, in which two monomer are shown in red and blue backbones, respectively, with transparent surface profiles. The sidechains that make physical contacts are highlighted with VDW representation. VDW, van der Waals.

The values of the optimized parameters for pairwise interactions of interfacial residues were highly dependent on the local secondary structural elements, suggesting that the secondary structural types at interfaces of protein complexes contribute differently to binding free energy. Among all parameters of pairwise interactions, we focused on the data that had the lowest values, which indicate significant contributions to binding affinity. We noticed that pairs of amino acids, which formed specific types of interactions, were often found in the list. For instance, the weights of the pair between Ile and Val as well as the one between Val and Leu showed very negative values that were averaged over all secondary structural types (Figure 5A), indicating that a hydrophobic effect plays an important role in regulating the stability of protein complexes. Likewise, the importance of electrostatic interactions for binding affinity is reflected by the negative values for the weights between amino acids with opposite charges [44], for instance, between Lys and Asp (Figure 5B). Surprisingly, different from these attractive interactions formed between residues of opposite charges, the weight of the Lys-Lys pair was found to have a very low value. The statistical analysis of a previous study made a similar observation [45], *i.e.*, positively charged residues have an average tendency to form pairwise contact with each other. In contrast, much lower propensities were found for the negatively charged amino acids to pair with each other. It has further been illustrated that the hydrophobic contacts in the extended configurations between the aliphatic sidechains of these positively charged residues are responsible for this pairing propensity (Figure 5C). Finally, interactions between a large hydrophobic residue and a charged residue are also found to affect the binding affinity. For example, a low value of weight was derived for the pair between Lys and Trp, because if a cationic sidechain (Lys or Arg) is placed next to an aromatic sidechain (Phe, Trp, or Tyr), the geometry will be biased toward one that would experience a favorable cation-π interaction [46] (Figure 5D).

The parameters for the non-interfacial residues were also found to be highly dependent on their local structural environments. This indicates that residues located in different parts of a protein complex cause different effects on binding stability, even when they are remote from the binding interface. Different from the weights of interfacial residues, however, no specific patterns can be recognized by the weights of non-interfacial residues. This is partially true because these non-pairwise terms are degenerated more by mixing various energetic factors. Nevertheless, through an unbiased and blind refinement process, our study showed that the searching was converged to a weight space in which the values of many parameters can be interpreted by basic physical and chemical properties. This demonstrates the effectiveness and reliability of our method in exploring the determinants of binding affinity.

## Conclusion

PPIs determine the functions of most cellular processes [47], [48], [49]. The stability of these interactions and their changes due to protein mutations are quantified by the measurement of binding affinity. However, most relevant experimental methods are either labor-intensive or time-consuming. This leaves a huge opportunity for *in silico* approaches that can predict binding affinity or mutation-induced affinity changes of PPIs. Based on the hypothesis that the stability of a protein complex is contributed by both the pairwise interactions of interfacial residues and all other non-interfacial residues, we reconstruct the calculation of binding affinity by decomposing it into the energetic contributions of both interfacial and non-interfacial residues in a protein complex. We further assume that the contributions of both interfacial and non-interfacial residues to the binding affinity depend on their local structural environments, such as secondary structural types and SASs. After refining the weights for all corresponding components against a large-scale dataset, we show that the model not only shows a strong correlation between the absolute values of the experimental and calculated binding affinities, but also can effectively predict the relative changes of binding affinities from mutations. Upon examining the values of weights in the optimized parameter space, we observe that many capture the chemical and physical principles of molecular recognition, which are not incorporated in a statistical potential derived from the structure of protein complexes. In summary, we demonstrate that our computational model reversely engineers the energetic components of protein complexes. Therefore, the proposed model can be a useful tool to predict binding affinity and understand the molecular mechanism of PPIs.

## Code availability

The source codes for cross-validation of binding affinity estimation against the SKEMPI dataset can be found in the GitHub repository: https://github.com/wulab-github/AffPred.

## Competing interests

The authors declare no conflict of financial interests.

### CRediT authorship contribution statement

**Bo Wang:** Methodology, Software, Data curation, Formal analysis, Validation. **Zhaoqian Su:** Data curation, Software, Validation. **Yinghao Wu:** Conceptualization, Methodology, Software, Supervision, Writing – original draft, Writing – review & editing, Funding acquisition.

## Supplementary material

Supplementary Table S1 **Estimate the confidence interval of our prediction results**

Supplementary Table S2 **The average values of optimized parameters for pairwise interactions of interfacial residues**

Supplementary Table S3 **The average values of optimized parameters for non-interfacial residues**
