# Supplementary material for: Computational Assessment of Protein–protein Binding Affinity by Reversely Engineering the Energetics in Protein Complexes
Source: Genomics Proteomics Bioinformatics. 2021 Apr 7;19(6):1012–22. doi: 10.1016/j.gpb.2021.03.004 (PMC9403033; doi:10.1016/j.gpb.2021.03.004)
Supplement: Supplementary Table S1 — Estimate the confidence interval of our prediction results [file mmc1.docx]

**Table S1** **Estimate the confidence interval of our prediction results**

| **Index** | **PCC for refinement** | **PCC for testing** |
| --- | --- | --- |
| 1 | 0.8397 | 0.6654 |
| 2 | 0.8398 | 0.6234 |
| 3 | 0.8362 | 0.6644 |
| 4 | 0.8389 | 0.6793 |
| 5 | 0.8399 | 0.6578 |
| 6 | 0.8394 | 0.6381 |
| 7 | 0.8359 | 0.6449 |
| 8 | 0.8352 | 0.6588 |
| 9 | 0.8376 | 0.6738 |
| 10 | 0.8374 | 0.6487 |

*Note*: The calculated PCCs during the refinement and during the testing for 10 runs of cross-validation. The average value of PCC is 0.66, and the standard deviation (SD) is 0.017.
