# Supplementary material for: Computational Assessment of Protein–protein Binding Affinity by Reversely Engineering the Energetics in Protein Complexes
Source: Genomics Proteomics Bioinformatics. 2021 Apr 7;19(6):1012–22. doi: 10.1016/j.gpb.2021.03.004 (PMC9403033; doi:10.1016/j.gpb.2021.03.004)
Supplement: Supplementary Table S2 — The average values of optimized parameters for pairwise interactions of interfacial residues [file mmc2.docx]

**Table S2** **The average values of optimized parameters for pairwise interactions of interfacial residues**

| **i j HH SS LL HL SL HS** |
| --- |
| 1 1 0.00000 -0.00217 0.66105 0.00000 -0.18715 -0.00043 |
| 2 1 0.62207 0.64103 0.00534 -0.02520 0.07841 0.00000 |
| 2 2 -0.06136 0.26490 -0.18781 0.64915 0.22777 0.31543 |
| 3 1 -0.00129 -0.54104 -0.13714 0.41186 0.38306 0.06820 |
| 3 2 -0.05381 0.25251 -0.03314 0.19428 0.20129 0.29155 |
| 3 3 0.00000 0.01248 -0.62148 -0.57608 -0.04919 -0.00340 |
| 4 1 0.00000 0.00000 0.14456 0.50605 0.10430 0.00000 |
| 4 2 -0.50739 0.13708 -0.14093 0.07462 0.20062 0.55740 |
| 4 3 0.00362 -0.06748 -0.53398 -0.23647 -0.38072 -0.09348 |
| 4 4 0.00000 0.00000 0.29339 -0.38667 -0.36146 0.13857 |
| 5 1 0.00850 -0.00364 -0.37067 0.26188 -0.48672 0.00000 |
| 5 2 -0.42791 -0.31564 -0.22737 0.50814 -0.08356 0.49279 |
| 5 3 0.00000 -0.38337 -0.43829 0.21182 -0.42176 -0.00619 |
| 5 4 0.12495 -0.35812 -0.07228 0.24381 -0.22919 0.01795 |
| 5 5 -0.37638 0.30198 0.01461 0.20556 0.11650 0.22656 |
| 6 1 0.00104 -0.11802 0.21993 0.36273 0.30466 0.00000 |
| 6 2 -0.07178 0.10081 -0.05485 0.16079 -0.08200 0.29562 |
| 6 3 0.35405 -0.11665 -0.03368 0.04029 0.54491 -0.00365 |
| 6 4 0.00651 0.37542 0.06000 0.03602 -0.41123 0.09516 |
| 6 5 -0.49722 0.15712 -0.31671 -0.27996 -0.31552 0.46142 |
| 6 6 0.00000 -0.37765 0.11852 0.46942 0.25529 0.21443 |
| 7 1 0.00000 0.04659 0.38687 0.53061 -0.44696 0.00000 |
| 7 2 0.00000 0.32523 -0.00457 0.21547 -0.18865 -0.00356 |
| 7 3 -0.00223 -0.09778 -0.09887 0.04788 0.24331 0.00000 |
| 7 4 0.00000 0.17535 0.52460 -0.45762 0.24185 0.01010 |
| 7 5 -0.00268 -0.01655 -0.15940 0.00162 -0.34598 -0.05491 |
| 7 6 0.00000 -0.01237 -0.36350 -0.11764 0.16329 0.00000 |
| 7 7 0.00000 -0.38371 -0.05230 0.47053 -0.14765 0.00000 |
| 8 1 -0.06361 0.00000 0.44558 -0.07570 0.42622 0.05739 |
| 8 2 0.00000 0.38565 0.27204 0.50321 0.02161 0.03505 |
| 8 3 -0.20379 -0.01785 -0.01021 0.36082 -0.02230 0.00000 |
| 8 4 0.04178 -0.32216 0.49616 0.39428 0.18454 0.34201 |
| 8 5 0.19738 0.08977 -0.18219 -0.13641 0.17310 0.03316 |
| 8 6 0.40453 -0.19526 0.07486 0.06247 0.07307 -0.00751 |
| 8 7 0.00000 0.33071 0.51374 0.50525 0.07800 0.00000 |
| 8 8 -0.02702 -0.16200 -0.24902 0.07811 -0.06951 0.31027 |
| 9 1 0.00000 -0.31449 0.11527 -0.25572 0.00030 0.02029 |
| 9 2 0.40832 -0.19344 0.43224 0.36887 0.19810 0.50541 |
| 9 3 -0.31227 -0.00835 -0.11837 0.42806 0.05083 -0.38142 |
| 9 4 0.09901 0.00000 0.12941 0.09190 -0.02117 0.00000 |
| 9 5 0.00000 0.02797 -0.03838 0.02282 0.03930 0.25478 |
| 9 6 -0.20785 0.02263 0.09421 0.03417 -0.07823 0.14023 |
| 9 7 -0.01824 0.08426 -0.09443 -0.03148 -0.00303 -0.35144 |
| 9 8 -0.22416 -0.10854 0.10205 -0.14144 -0.00237 -0.25203 |
| 9 9 -0.02191 0.00000 0.21789 -0.18533 0.02650 0.00000 |
| 10 1 0.37211 0.00000 0.25457 0.07779 0.01726 0.00000 |
| 10 2 0.35562 0.12219 0.34591 0.28752 -0.21923 0.28819 |
| 10 3 -0.03408 0.00000 0.33727 0.16981 -0.14939 0.00000 |
| 10 4 0.00000 0.07529 0.33742 0.31713 0.00287 0.00237 |
| 10 5 0.10526 0.00000 -0.25661 0.16659 0.11741 0.00000 |
| 10 6 0.06453 0.00000 0.25479 -0.05512 0.04369 -0.05212 |
| 10 7 -0.02524 0.48423 0.33345 0.01361 0.29026 -0.11641 |
| 10 8 0.03722 0.00000 0.20458 0.00029 0.18448 -0.24960 |
| 10 9 0.00000 0.01753 -0.00118 -0.02553 -0.07868 -0.03280 |
| 10 10 0.46333 0.00000 0.29385 -0.18439 0.15240 -0.08902 |
| 11 1 0.00000 -0.07693 0.06510 0.08443 0.27204 0.00000 |
| 11 2 0.06873 0.19327 0.04311 0.10560 -0.07321 0.10987 |
| 11 3 0.00000 -0.08743 -0.09703 -0.14274 -0.01265 0.01718 |
| 11 4 -0.18472 -0.42383 0.11832 -0.15544 -0.20706 0.00000 |
| 11 5 -0.20730 -0.25794 -0.00115 -0.19799 0.17812 -0.00308 |
| 11 6 -0.26087 0.07382 0.10784 -0.01280 0.17138 0.12098 |
| 11 7 0.25834 0.08615 0.05774 0.02857 0.02320 -0.01331 |
| 11 8 0.05266 0.06210 0.06614 0.08109 -0.04856 -0.15331 |
| 11 9 0.01612 0.20773 0.11459 -0.07836 -0.07687 0.07440 |
| 11 10 -0.26364 -0.14126 0.26053 0.01030 0.15887 -0.00338 |
| 11 11 0.00000 0.01174 0.34942 -0.32515 -0.23322 -0.13130 |
| 12 1 0.00000 -0.42721 -0.10502 -0.03388 0.11468 -0.23561 |
| 12 2 0.51229 0.12246 0.02929 0.31945 0.11283 0.41001 |
| 12 3 0.02166 0.35587 0.16706 -0.22481 -0.28120 0.00000 |
| 12 4 0.17120 0.32676 0.04134 -0.07882 -0.32248 -0.21694 |
| 12 5 -0.10779 0.17593 0.19399 0.24750 -0.40570 0.05150 |
| 12 6 -0.13955 0.11361 0.08022 -0.02098 -0.02500 -0.10164 |
| 12 7 -0.30384 0.06051 0.28635 -0.23099 0.04313 -0.08260 |
| 12 8 -0.34459 0.04343 -0.21840 -0.05430 -0.09770 -0.07999 |
| 12 9 -0.03712 0.36587 0.19419 0.04751 -0.04504 -0.20287 |
| 12 10 -0.26263 0.07945 -0.33746 -0.03218 -0.07738 -0.01368 |
| 12 11 -0.27547 -0.10906 -0.37382 -0.04012 -0.07848 -0.10956 |
| 12 12 -0.19811 -0.06506 -0.22799 -0.45961 -0.25611 -0.12405 |
| 13 1 0.00000 0.00365 0.30123 0.01047 0.27463 0.00000 |
| 13 2 0.12093 -0.02587 0.14279 0.25812 0.50458 0.25876 |
| 13 3 -0.15210 -0.06203 0.15162 -0.06075 0.12716 -0.06075 |
| 13 4 -0.24445 0.00000 -0.03480 0.22264 -0.01544 0.15218 |
| 13 5 -0.11322 -0.29550 0.31093 -0.13819 -0.21910 -0.27936 |
| 13 6 0.23918 -0.09994 0.20878 0.00402 0.20352 -0.05769 |
| 13 7 -0.00150 0.04266 -0.02573 -0.07180 0.27754 -0.11833 |
| 13 8 -0.18307 0.04087 0.26700 -0.06267 -0.02003 -0.21900 |
| 13 9 0.05388 0.03909 0.16101 0.07377 0.02359 -0.08935 |
| 13 10 -0.23752 -0.07207 0.00482 -0.04060 0.22449 -0.13527 |
| 13 11 0.33021 -0.13881 -0.23140 -0.05553 -0.06621 -0.03895 |
| 13 12 -0.26141 -0.07992 -0.02919 0.05164 0.07005 0.00492 |
| 13 13 -0.10803 -0.09522 -0.03854 0.06132 -0.19127 0.13022 |
| 14 1 0.00000 0.19920 0.03694 0.08029 0.06721 0.00000 |
| 14 2 -0.35649 0.00000 -0.25108 0.00000 -0.19057 0.28708 |
| 14 3 0.00000 -0.32024 0.06849 -0.30852 0.04777 0.00000 |
| 14 4 0.00000 0.00000 -0.01805 -0.07883 0.16033 0.00000 |
| 14 5 0.00000 -0.16579 -0.10289 -0.16389 -0.13078 -0.01073 |
| 14 6 0.11088 0.00000 0.16327 0.16064 0.13151 0.14413 |
| 14 7 0.00000 0.00000 0.41572 -0.18306 0.39784 0.00000 |
| 14 8 0.00000 0.00000 0.43185 0.23602 0.40464 0.00000 |
| 14 9 0.00000 0.18908 0.04127 -0.09878 0.14249 0.00000 |
| 14 10 0.00000 0.00000 0.25787 0.08884 0.30411 0.00000 |
| 14 11 0.00000 0.00000 0.07332 0.05615 0.38538 -0.05087 |
| 14 12 0.11147 -0.00755 -0.35062 -0.06520 0.10890 0.02549 |
| 14 13 0.02018 -0.02949 0.11510 0.43506 0.14000 -0.01606 |
| 14 14 0.00129 0.00000 0.28902 0.00000 -0.20215 0.00000 |
| 15 1 0.00000 0.00000 -0.31325 -0.05733 -0.22023 0.00000 |
| 15 2 0.00000 0.00000 -0.24486 -0.02083 -0.24849 -0.35621 |
| 15 3 0.00000 -0.31986 -0.13045 -0.15090 -0.18399 -0.05968 |
| 15 4 0.00000 0.00000 -0.10953 0.25804 0.14378 0.01216 |
| 15 5 0.00000 0.09224 -0.06339 -0.08975 -0.21265 -0.23273 |
| 15 6 0.00000 -0.01159 -0.21152 -0.18367 -0.24316 0.45347 |
| 15 7 0.00000 0.00000 -0.07869 -0.18980 -0.32238 -0.43787 |
| 15 8 0.00000 0.00000 0.10327 0.28702 0.06323 -0.25395 |
| 15 9 0.00000 -0.03200 -0.05370 0.24504 0.04472 0.16741 |
| 15 10 0.00000 -0.17076 -0.20261 -0.16140 0.12832 0.31908 |
| 15 11 0.00000 0.38005 0.17458 0.11019 0.23936 -0.35119 |
| 15 12 -0.09352 0.00000 0.38242 -0.17391 0.37961 -0.24312 |
| 15 13 0.00000 -0.28512 0.08887 0.03929 0.22018 -0.07615 |
| 15 14 0.00000 0.00000 -0.05663 -0.29660 -0.08910 -0.18662 |
| 15 15 0.00000 0.00000 0.00000 -0.34591 -0.37413 -0.43745 |
| 16 1 0.00000 0.22148 -0.14451 -0.13524 -0.22549 0.00000 |
| 16 2 0.00000 0.20404 -0.00822 0.17264 -0.16385 0.17507 |
| 16 3 -0.15287 0.13340 0.02660 -0.29982 -0.22272 -0.00556 |
| 16 4 -0.11695 0.04887 0.07036 0.03827 -0.04084 0.07031 |
| 16 5 -0.18567 0.06073 -0.07812 -0.32992 -0.04071 -0.04590 |
| 16 6 0.00000 0.28491 0.03209 -0.22986 -0.16640 -0.10522 |
| 16 7 0.00000 0.01133 -0.00228 -0.27848 -0.21504 -0.05144 |
| 16 8 0.00000 0.23045 0.28093 -0.00648 -0.24099 -0.07313 |
| 16 9 -0.31302 0.31860 0.00211 0.10665 -0.09006 0.27932 |
| 16 10 0.00000 0.34742 0.00628 0.05246 0.13132 0.04399 |
| 16 11 -0.02786 0.13542 0.08708 -0.00233 -0.04323 -0.11041 |
| 16 12 0.23526 0.12656 0.27346 0.02487 0.18170 0.08535 |
| 16 13 0.00000 0.02096 -0.01678 -0.07848 -0.02500 0.00306 |
| 16 14 0.00000 -0.01254 0.04784 -0.11491 -0.27208 -0.00122 |
| 16 15 0.00335 0.21853 0.16986 -0.35388 0.04317 -0.14618 |
| 16 16 0.00000 -0.01669 0.04568 -0.08423 -0.08366 -0.03195 |
| 17 1 0.05586 0.02023 -0.12661 -0.23632 -0.18848 0.20003 |
| 17 2 0.36463 0.24146 -0.02757 0.29510 -0.19623 0.30187 |
| 17 3 0.08407 0.04255 -0.02247 -0.00062 -0.24954 -0.02697 |
| 17 4 0.00000 -0.19001 -0.32517 -0.06581 -0.07412 0.10213 |
| 17 5 0.12525 0.18057 0.07401 -0.30137 -0.08455 -0.12835 |
| 17 6 -0.00027 0.00597 -0.02271 -0.15696 -0.14276 0.06767 |
| 17 7 0.00000 -0.20664 -0.18339 -0.12878 -0.23175 -0.25279 |
| 17 8 0.00000 -0.00045 -0.01704 0.00519 0.02715 -0.00871 |
| 17 9 -0.07802 -0.03176 -0.17857 -0.05560 -0.02404 0.01829 |
| 17 10 -0.00027 -0.04374 -0.01161 -0.04409 0.06308 -0.11818 |
| 17 11 0.00000 0.06683 0.01205 0.01515 -0.17189 -0.26877 |
| 17 12 0.01949 0.01872 -0.01059 -0.01958 -0.22842 -0.08912 |
| 17 13 0.03248 -0.00938 0.07490 0.21078 -0.02649 -0.01962 |
| 17 14 0.00000 0.00000 -0.17876 -0.08638 -0.15685 -0.10303 |
| 17 15 0.00000 0.03849 -0.01267 -0.01016 -0.19626 -0.28723 |
| 17 16 0.00000 -0.10944 -0.24504 -0.21298 0.08687 -0.03955 |
| 17 17 0.00000 -0.03176 -0.00070 -0.19442 -0.24659 0.20483 |
| 18 1 0.00000 -0.12034 0.14395 -0.04540 -0.00144 0.07854 |
| 18 2 0.00000 0.40456 0.05864 0.25886 0.04733 -0.00813 |
| 18 3 -0.26541 -0.11217 -0.00115 -0.03444 0.05615 0.15093 |
| 18 4 -0.16504 0.17878 0.10839 -0.04540 -0.16125 0.08488 |
| 18 5 0.00000 0.06239 -0.11947 -0.02500 -0.01113 -0.24647 |
| 18 6 0.00000 -0.21953 -0.00578 -0.17085 -0.13338 -0.25099 |
| 18 7 0.00000 0.09409 0.09773 -0.00213 -0.26875 0.06883 |
| 18 8 0.00000 0.02710 0.24592 0.00569 -0.14417 0.01183 |
| 18 9 0.00000 0.09712 -0.04226 -0.17445 -0.06328 -0.18010 |
| 18 10 0.00000 0.00000 0.05437 -0.13542 0.04688 0.11998 |
| 18 11 0.00000 -0.08105 -0.01517 -0.01537 -0.16350 -0.12526 |
| 18 12 -0.22413 -0.15336 -0.28263 -0.04000 0.07345 -0.20122 |
| 18 13 -0.00268 0.00257 -0.05498 -0.03464 0.02997 -0.01273 |
| 18 14 0.00000 0.00000 0.01165 -0.07430 0.07450 -0.26395 |
| 18 15 0.00000 0.34421 -0.32962 -0.26965 -0.20163 0.26414 |
| 18 16 0.00000 0.02117 0.02671 -0.09441 0.06163 -0.12650 |
| 18 17 0.00000 -0.06212 -0.03739 -0.28508 -0.00595 -0.11351 |
| 18 18 0.00000 -0.18984 -0.00742 -0.31105 -0.04245 -0.15619 |
| 19 1 0.00000 0.00000 -0.05762 0.00780 0.06817 0.00000 |
| 19 2 0.00000 0.27083 0.28555 -0.00351 0.05933 0.09300 |
| 19 3 0.17843 0.00000 -0.05445 0.09809 -0.10903 0.00000 |
| 19 4 0.01629 0.00000 0.00574 0.08852 -0.01696 0.00000 |
| 19 5 -0.11102 0.00000 0.00826 -0.03613 -0.13243 -0.05786 |
| 19 6 0.00000 -0.01071 0.18992 -0.09947 0.29030 0.07472 |
| 19 7 0.00000 0.20593 -0.20875 -0.09676 -0.35577 0.00000 |
| 19 8 0.00000 -0.18331 0.24409 -0.19000 0.19043 0.09074 |
| 19 9 0.14555 0.33548 -0.07954 -0.00988 -0.11856 -0.09424 |
| 19 10 0.00000 -0.26043 -0.01404 -0.10672 -0.01846 0.00000 |
| 19 11 0.00000 -0.07866 0.10756 -0.35661 -0.00785 0.20259 |
| 19 12 -0.10881 0.21721 -0.20211 0.11886 0.24601 0.22949 |
| 19 13 -0.04113 0.00000 -0.14202 0.04073 0.23544 -0.08967 |
| 19 14 0.00000 0.00000 -0.04001 -0.27909 -0.06568 0.00000 |
| 19 15 0.00000 0.03685 0.32039 -0.10516 -0.20321 -0.25355 |
| 19 16 -0.13616 0.07903 0.11489 -0.07498 0.08131 -0.21460 |
| 19 17 0.00000 -0.10342 -0.12235 -0.17791 -0.11695 -0.25381 |
| 19 18 0.00000 0.00000 0.13673 0.08502 0.02354 0.13780 |
| 19 19 0.00000 0.00000 0.21428 -0.11904 0.09647 0.00000 |
| 20 1 0.00000 0.00000 -0.05772 0.01217 0.28938 0.00000 |
| 20 2 0.00000 0.19292 -0.10709 0.19920 0.18139 0.00000 |
| 20 3 0.00000 0.15218 0.15121 -0.07792 0.15192 0.00000 |
| 20 4 0.00106 0.00000 0.06929 0.05978 -0.15608 0.00000 |
| 20 5 0.00000 0.05262 -0.07048 -0.12997 -0.15764 0.00000 |
| 20 6 0.00000 0.01870 0.11435 0.15655 -0.20691 0.01721 |
| 20 7 -0.17800 0.11039 0.02549 0.15395 -0.05530 0.21247 |
| 20 8 0.00000 0.00000 0.09377 0.00761 0.32579 0.00000 |
| 20 9 0.00000 0.15846 0.12792 -0.04714 0.07357 -0.00410 |
| 20 10 0.00000 0.00000 -0.01502 -0.12087 0.14760 0.21245 |
| 20 11 0.00000 0.00000 0.09628 -0.25475 0.02285 0.00000 |
| 20 12 0.00000 0.00000 0.21887 -0.04688 0.20442 -0.29015 |
| 20 13 0.01079 0.00000 0.20039 -0.24772 0.11584 -0.01958 |
| 20 14 0.00000 0.00000 0.16497 -0.28834 0.25383 0.00000 |
| 20 15 0.00000 0.00000 -0.01351 -0.10013 0.05673 -0.30805 |
| 20 16 0.00000 0.05569 0.01219 -0.16245 0.08845 0.00000 |
| 20 17 -0.00509 0.03898 -0.04539 0.11542 -0.03030 -0.09367 |
| 20 18 0.00000 0.00000 -0.25225 0.12863 0.09463 0.00000 |
| 20 19 0.00000 0.00000 -0.02641 0.35424 0.26717 -0.13256 |
| 20 20 0.00000 0.06811 0.22730 -0.33870 -0.03495 0.00000 |

*Note*: All the possible pairwise combinations of 20 amino acid types are indicated by the first two columns on the left (index i and j), in which 1 is GLY; 2 is ALA; 3 is VAL; 4 is ILE; 5 is LEU; 6 is SER; 7 is THR; 8 is ASP; 9 is ASN; 10 is GLU; 11 is GLN; 12 is LYS; 13 is ARG; 14 is CYS; 15 is MET; 16 is PHE; 17 is TYR; 18 is TRP; 19 is HIS; and 20 is PRO. The next six columns give the secondary structural combinations, in which HH stands for helix-helix interactions; SS stands for strand-strand interactions; LL stands for loop-loop interactions; HL stands for helix-loop interactions; SL stands for strand-loop interactions; and HS stands for helix-loop interactions.
