# Supplementary material for: Computational Assessment of Protein–protein Binding Affinity by Reversely Engineering the Energetics in Protein Complexes
Source: Genomics Proteomics Bioinformatics. 2021 Apr 7;19(6):1012–22. doi: 10.1016/j.gpb.2021.03.004 (PMC9403033; doi:10.1016/j.gpb.2021.03.004)
Supplement: Supplementary Table S3 — The average values of optimized parameters for non-interfacial residues [file mmc3.docx]

**Table S3** **The average values of optimized parameters for non-interfacial residues**

| **Index H-S S-S L-S H-I S-I L-I** |
| --- |
| 1 0.10843 -0.20504 0.09990 0.05021 0.53099 -0.29126 |
| 2 -0.25799 -0.03699 0.13696 -0.10741 -0.33141 -0.06421 |
| 3 0.53116 -0.08747 -0.23890 0.03934 0.08902 -0.14739 |
| 4 -0.72883 0.21291 -0.51116 0.34317 0.30982 0.00187 |
| 5 0.25737 0.28799 0.23435 0.02803 -0.23871 -0.04369 |
| 6 -0.42128 0.28766 -0.34659 0.68870 -0.19645 -0.05859 |
| 7 -0.77920 -0.04804 -0.17960 0.26138 0.12542 0.04055 |
| 8 0.08864 0.06736 0.49578 0.31691 0.30402 0.25068 |
| 9 0.34674 -0.47007 -0.14266 0.79124 0.52736 0.22291 |
| 10 -0.25792 -0.39633 0.15822 0.43453 -0.65503 0.45560 |
| 11 0.23280 0.22633 -0.30004 -0.05815 -0.14668 -0.25092 |
| 12 0.17721 0.03286 -0.31987 -0.08103 -0.19700 -0.72947 |
| 13 0.05928 -0.23611 -0.06565 0.07415 0.09086 -0.48295 |
| 14 0.58431 0.64522 0.46680 -0.40823 0.00125 -0.00781 |
| 15 -0.42060 -0.59629 -0.35171 -0.34677 0.06768 -0.59142 |
| 16 0.68590 0.54551 -0.28617 -0.33268 0.67242 0.54865 |
| 17 0.43919 -0.13707 0.22890 0.03254 -0.09420 0.33607 |
| 18 0.38923 0.25658 -0.75969 -0.30222 -0.03248 -0.74853 |
| 19 -0.26471 -0.33492 -0.00625 -0.54202 -0.76655 -0.30159 |
| 20 -0.22206 -0.01704 0.29624 -0.35516 0.32658 0.31767 |

*Note*: All the 20 amino acid types are indicated by the first columns on the left, following the same index as in Table S2. While the next column H-S means the secondary structure of the residue is helix (H) is it is on the surface (S). Similarly, the definitions in the next five columns are as follows: S-S means the secondary structure of the residue is strand is it is on the surface; L-S means the secondary structure of the residue is loop is it is on the surface; H-I means the secondary structure of the residue is helix is it is at the interior; S-I means the secondary structure of the residue is strand is it is at the interior; L-I means the secondary structure of the residue is loop is it is at the interior.
